# P02.90. Equivalence of doctor interactions between Activator Methods and sham chiropractic protocols during an expertise-based randomized clinical trial

**DOI:** 10.1186/1472-6882-12-S1-P146

**Published:** 2012-06-12

**Authors:** J DeVocht, S Salsbury, M Seidman, L Carber, W Schaeffer, C Stanford, C Goertz, M Spector, M Hondras

**Affiliations:** 1 Palmer College of Chiropractic, Davenport, USA; 2 Private Practice, Coralville, USA; 3 University of Iowa, Iowa City, USA

## Purpose

One objective of the expertise-based randomized controlled trial portion of a current developmental center grant is to determine which of three control groups would be most appropriate for a larger scale study concerning the effectiveness of Activator Methods chiropractic technique (AMCT) for temporomandibular disorders. A video evaluation instrument was developed to assess the equivalence of doctor interactions with participants in the active and sham AMCT groups.

## Methods

One doctor of chiropractic (DC) delivered the chiropractic intervention to the active and sham AMCT groups while being video recorded. The evaluation instrument codified DC communications into 4 domains: therapeutic (information seeking, explanations), procedural (directions, cautions, logistics), effectiveness (optimistic, pessimistic, neutral), and affective (social, name use) interactions. Activator Adjusting Instrument (AAI) clicks, encounter duration, touch orientation, and evaluator assessment of treatment group were documented. A trained video evaluator, blinded to treatment group, coded 34 active and 30 sham treatment videos by placing a hash mark in the appropriate category for each interaction. Descriptive statistics included medians and interquartile ranges.

## Results

DC-initiated verbal communications were similar between active and sham AMCT in the procedural and affective domains. Notable differences were observed in the medians of the number of DC-initiated verbal communications between active and sham AMCT sessions in the therapeutic and effectiveness domains. More AAI clicks were recorded for active (42) vs sham (22) AMCT. Encounter duration also differed between active and sham AMCT (13 vs 11 minutes). The video evaluator correctly identified 66% of active AMCT, but only 31% of sham sessions.

## Conclusion

Definitive conclusions about how differences in DC behaviors may have impacted study results cannot be drawn until we have completed data analysis for the primary endpoint. Investigators may want to consider adding this type of analysis in manual therapies when sham or other control groups are used.

